# Barriers to the utilization of community-based child and newborn health services in Ethiopia: a scoping review

**DOI:** 10.1093/heapol/czab047

**Published:** 2021-05-27

**Authors:** Nathan P Miller, Farid Bagheri Ardestani, Hayes Wong, Sonya Stokes, Birkety Mengistu, Meron Paulos, Nesibu Agonafir, Mariame Sylla, Agazi Ameha, Bizuhan Gelaw Birhanu, Sadaf Khan, Ephrem Tekle Lemango

**Affiliations:** Health Section, UNICEF, 3 UN Plaza, New York, NY 10017, USA; Department of Population and Family Health, Mailman School of Public Health, Columbia University, 722 West 168th St., New York, NY 10032, USA; Health Section, UNICEF, 3 UN Plaza, New York, NY 10017, USA; Department of Population and Family Health, Mailman School of Public Health, Columbia University, 722 West 168th St., New York, NY 10032, USA; Department of Population and Family Health, Mailman School of Public Health, Columbia University, 722 West 168th St., New York, NY 10032, USA; PATH Ethiopia, Bole Medhaniyalem Street #03, Bole, Addis Ababa, Ethiopia; PATH Ethiopia, Bole Medhaniyalem Street #03, Bole, Addis Ababa, Ethiopia; PATH Ethiopia, Bole Medhaniyalem Street #03, Bole, Addis Ababa, Ethiopia; Health Section, UNICEF Ethiopia, UNECA Compound, Zambezi Building, Addis Ababa, Ethiopia; Health Section, UNICEF Ethiopia, UNECA Compound, Zambezi Building, Addis Ababa, Ethiopia; Health Section, UNICEF Ethiopia, UNECA Compound, Zambezi Building, Addis Ababa, Ethiopia; Maternal Newborn Child Health and Nutrition, PATH, 2201 Westlake Ave. Ste 200, Seattle, WA 98121, USA; Programs Section, Maternal, Child Health and Nutrition Directorate, Ministry of Health, Sudan Street, Addis Ababa, Ethiopia

**Keywords:** Care seeking, community health, Ethiopia, health systems, newborn and child health, utilization

## Abstract

The Ethiopian Federal Ministry of Health and partners have scaled up integrated community case management (iCCM) and community-based newborn care (CBNC), allowing health extension workers (HEWs) to manage the major causes of child and newborn death at the community level. However, low service uptake remains a key challenge. We conducted a scoping review of peer-reviewed and grey literature to assess barriers to the utilization of HEW services and to explore potential solutions. The review, which was conducted to inform the Optimizing the Health Extension Program project, which aimed to increase the utilization of iCCM and CBNC services, included 24 peer-reviewed articles and 18 grey literature documents. Demand-side barriers to utilization included lack of knowledge about the signs and symptoms of childhood illnesses and danger signs; low awareness of curative services offered by HEWs; preference for home-based care, traditional care, or religious intervention; distance, lack of transportation and cost of care seeking; the need to obtain husband’s permission to seek care and opposition of traditional or religious leaders. Supply-side barriers included health post closures, drug stockouts, disrespectful care and limited skill and confidence of HEWs, particularly with regard to the management of newborn illnesses. Potential solutions included community education and demand generation activities, finding ways to facilitate and subsidize transportation to health facilities, engaging family members and traditional and religious leaders, ensuring consistent availability of services at health posts and strengthening supervision and supply chain management. Both demand generation and improvement of service delivery are necessary to achieve the expected impact of iCCM and CBNC. Key steps for improving utilization would be carrying out multifaceted demand generation activities, ensuring availability of HEWs in health posts and ensuring consistent supplies of essential commodities. The Women’s Development Army has the potential to improving linkages between HEWs and communities, but this strategy needs to be strengthened to be effective.

**Key messages**

- The major barrier to the impact of community case management and community-based newborn care in Ethiopia is low utilization of services.
- There are a number of demand- and supply-side barriers to utilization.
- Improved demand generation and community engagement, as well as improved availability of services and drugs, are needed to increase utilization.
- Linkages between communities and health extension workers need to be improved to increase access to health services and to strengthen community engagement and education.

## Introduction

Ethiopia was one of 12 low-income countries to achieve the Millennium Development Goals target of reducing the under-five mortality rate by two-thirds between 1990 and 2015. The under-five mortality rate has fallen from 202 deaths among children under five per 1000 live births in 1990 to 55 per 1000 in 2018. Despite this remarkable achievement, there are still 191 000 under-five deaths per year in Ethiopia (UNICEF, 2019). The majority of these deaths are caused by pneumonia (17%), diarrhoea (8%) and neonatal causes (47%), with malnutrition as an important underlying factor (UNICEF and WHO, 2015). Ethiopia has also reduced the maternal mortality from 1250 maternal deaths per 100 000 live births in 1990 to 401 deaths per 100 000 live births in 2017 (WHO, 2019).

The Health Extension Program (HEP) is the flagship programme of the Ethiopian government to improve access to essential healthcare services. Within the HEP, health extension workers (HEWs) provide a large package of preventive, promotive and curative services. There are typically two HEWs based in a health post that serves a population of about 5000 people. Following a change in policy that allowed HEWs to treat pneumonia, the Federal Ministry of Health (FMOH), with support from implementing partners, began scaling up integrated community case management (iCCM) in 2010, followed by community-based newborn care (CBNC)—for which HEWs were trained to carry out prenatal and postnatal contacts with mothers and newborns and to identify and treat newborns with signs of possible severe bacterial infection—in 2013. With the iCCM and CBNC initiatives, HEWs manage the major causes of child and newborn death at the community level. In 2010, the Ethiopian government introduced the Women’s Development Army (WDA) to help bridge the gap between communities and HEWs. The plan for the WDA was to train one woman out of every five households to serve as an unpaid community-based health cadre who would carry out community education and mobilization activities and serve as the HEWs’ voices in communities (Yitbarek *et al.*, 2019).

Routine data and independent evaluations of the scale-up of iCCM showed that the strength of programme implementation (training, supervision and supply of commodities) was strong and the quality of care provided by HEWs was relatively high, but care seeking and utilization of iCCM services were very low, particularly for children under 2 months of age (Miller *et al.*, 2014; Tadesse *et al.*, 2014; Amouzou *et al.*, 2016b; Doherty *et al.*, 2014). The 2016 Ethiopia Demographic and Health Survey found that care seeking for under-five children in rural areas for pneumonia, diarrhoea and fever were 31%, 44% and 35%, respectively (Central Statistical Agency [Ethiopia] and ICF International, 2017). The utilization of services provided by HEWs for sick children did seem to increase after the introduction of iCCM (Miller *et al.*, 2014), and iCCM utilization rates and care seeking for sick children increased after sustained implementation over time (Tadesse *et al.*, 2014; Ashenafi *et al.*, 2014). Likewise, the utilization of HEW services for newborns increased after the implementation of CBNC (Ameha *et al.*, 2019). However, despite these increases, care seeking from HEWs for sick children still remained relatively rare (Ashenafi *et al.*, 2014; Amouzou *et al.*, 2016a).

Given the large investments in iCCM and CBNC and the importance of increasing community utilization of these services, the Bill and Melinda Gates Foundation supported the authors’ organizations to implement the Optimizing the Health Extension Program (OHEP) project to increase the utilization of iCCM and CBNC services. This was a 3-year effort (March 2016–December 2018) to increase the uptake of iCCM and CBNC services in low-performing districts of Amhara; Oromia; Southern Nations, Nationalities and Peoples (SNNP); and Tigray Regions. The first step of this project was to review the existing literature to understand the evidence on barriers to the utilization of HEW services and to identify potentially effective interventions to increase the utilization of HEWs. Findings from this study were used to identify and implement evidence-based and context-specific interventions to address the complex factors influencing the low utilization of HEW services.

## Methods

### Research questions

Primary research question: What are the primary barriers to the utilization of health and nutrition services for children between 0 and 5 years of age provided by HEWs in Ethiopia?

Secondary research question: What are the promising interventions for increasing the utilization of health or nutrition services for children between 0 and 5 years of age provided by HEWs in Ethiopia?

### Search strategy

An initial literature review was conducted in 2015 to inform the design of the OHEP project. The review was then updated for this publication. The PubMed, Embase, Scopus and Global Health Ovid databases were searched on February 22, 2018, using the search terms in Supplementary Appendix 1. Results were filtered for articles published on or after January 1, 2010 (the first year of implementation of iCCM in Ethiopia).

In addition to the search of published literature, we also requested any relevant reports related to the utilization of or care seeking from HEWs from the Ethiopian FMOH, UN agencies, and non-governmental organizations known to support HEW services in Ethiopia.

### Screening of abstracts and full texts

All abstracts were screened for inclusion criteria by two researchers. For grey literature reports that did not contain abstracts or executive summaries, full reports were screened. Following the abstract screening, the full texts of the retained articles/reports were screened by two researchers. Any inconsistencies in inclusion/exclusion decisions between the two researchers were discussed until consensus was reached.

### Inclusion criteria

Articles/reports were included if they met *all* of the following criteria: referred to information from Ethiopia; was a research article or report; contained data on barriers to the utilization of or care seeking from HEWs/health post OR contained data on levels of utilization of or care seeking from HEWs/health post OR contained data on interventions to increase the utilization of or care seeking from HEWs/health post; referred to any health or nutrition services provided by HEWs/health post for children of 0–5 years of age; and reported a study that was conducted in 2010 or later.

### Data extraction and identification of themes

Full texts of journal articles and grey literature that were retained following the full-text screening were analysed for content relevant to the research questions. Relevant text was extracted using a standardized data extraction form and categorized into three main themes: (1) levels of utilization or care seeking, (2) barriers and facilitators of utilization and (3) interventions to improve utilization. Within the barriers and facilitators theme, text was divided into demand- and supply-side barriers and facilitators. Interventions to improve utilization were also categorized as demand- and supply-side interventions.

### Data synthesis

The extracted text was analysed to identify the main themes. Two researchers coded an initial sample of extracted text and compared the identified codes. Consensus was reached on the codes to be applied to the remaining text and a codebook was developed. Additional codes that were identified during analysis were added to the codebook. Once all text was coded, the main themes were identified. The text was analysed using the deductive framework described above. Within this framework, sub-themes were determined inductively based on the themes that appeared in the data.

Given that the objective of this review was to include the full range of relevant literature, including both quantitative and qualitative studies with a wide variety of methods, quality assessment of included literature was not carried out (Khalil *et al.*, 2016). Furthermore, because of the wide range of research questions and interventions described, the wide range of study methods applied in the literature, and the qualitative focus of the research questions of this scoping review, no assessment of the effectiveness of interventions was conducted.

## Results

### Search results

The peer-reviewed literature search yielded 1062 non-duplicative results. After screening of abstracts, 55 articles were retained for full-text screening. An additional 31 grey literature documents were provided by the FMOH and partners in Ethiopia. After screening of full texts, 24 peer-reviewed articles and 18 grey literature documents were included in the final analysis. Supplementary Appendix 2 presents the flowchart for the peer-reviewed and grey literature search process.

### Demand-side barriers

#### Knowledge and beliefs about childhood illness

A lack of knowledge about signs and symptoms of childhood illnesses and about danger signs was an important factor limiting care seeking. Qualitative and quantitative studies in Amhara, Benishangul-Gumuz, Oromia, SNNP and Tigray found incomplete knowledge of signs of childhood illnesses and danger signs (Tefera *et al.*, 2014a,b; Amare *et al.* 2013, Ethiopia Health and Nutrition Research Institute, 2013; Callaghan-Koru *et al.*, 2013; Sibley *et al.*, 2017; Mitiku and Assefa, 2017). This lack of knowledge meant that caregivers did not always recognize when a child needed care or delayed in seeking care (Shaw *et al.*, 2017; Demissie *et al.*, 2014; Kolola *et al.*, 2016; Sibley *et al.*, 2017; Gebre *et al.*, 2018; Mitiku and Assefa, 2017; Ethiopia Health and Nutrition Research Institute, 2013; Okwaraji *et al.*, 2017; Jembere *et al.*, 2017). Some caregivers also feared that young children were too fragile to withstand medicines (Tefera *et al.*, 2014b; Save the Children, 2013b)

In some cases, traditional beliefs discouraged caregivers from seeking formal care (The Last Ten Kilometers Project, 2013; Save the Children, 2015a; Shaw *et al.*, 2017; Sibley *et al.*, 2017; Jembere *et al.*, 2017). This was because of traditional beliefs of causes of illness, such as supernatural forces or sensitivity to sun, wind or cold (Tefera *et al.*, 2014a,b; Amare *et al.* 2013, Save the Children, 2015a, 2013b; Warren, 2010; Sisay *et al.*, 2014; Sibley *et al.*, 2017; Jembere *et al.*, 2017). There was also a high value placed on traditional ceremonies that involved prayer or use of holy water to treat children. These ceremonies could be performed as a part of home-based care but were not part of facility-based care (Save the Children, 2015a; Tegegne and Mersha, 2017). Caregivers also reported not seeking care, because they thought that the disease would resolve by itself over time (Gelaw *et al.*, 2014).

Traditional beliefs were particularly important in the case of newborn children and infants. Many communities believed that newborns should not be taken out of the house for any reason and that the child should be secluded from individuals outside the immediate family for the one to three months after birth (Doherty *et al.*, 2014; Save the Children, 2013b; Warren, 2010). Mothers in these communities faced social stigma if they took their newborns out of the home (Tefera *et al.*, 2014b). It was believed that the child would become ill if he/she left the home or was seen by outsiders (Save the Children, 2015b). In southeast Ethiopia, mothers reported a fear of side effects as a reason for not immunizing or not completing immunizations for their children (Legesse and Dechasa, 2015). Many also believe that the fate of young children was in God’s hands and that there was nothing to be done to help the child (Sisay *et al.*, 2014; Save the Children, 2015b). These traditional beliefs were especially strong among older generations (Sisay *et al.*, 2014).

#### Lack of awareness of services

A major reason for not using HEW services was a lack of awareness of the services offered or of the benefits of services, among communities (The Last Ten Kilometers Project, 2013; Save the Children, 2015a,b; Shaw *et al.*, 2015; Kelbessa *et al.*, 2014; Negussie and Girma, 2017; Sibley *et al.*, 2014; Okwaraji *et al.*, 2017; Jembere *et al.*, 2017). A survey in Amhara, Oromia, SNNP and Tigray in December 2012 found that 60% of caregivers were aware of curative services at the health post. Of those who knew about curative services, 80% knew about malaria treatment, 64% knew about diarrhoea treatment, 55% knew about treatment for acute respiratory infection and only 9% knew about malnutrition services (The Last Ten Kilometers Project, 2013). One survey in Oromia in 2013 found that only 43% of caregivers interviewed were aware of the availability of treatments for childhood illness at the health post (Shaw *et al.*, 2015). Lack of awareness of services was also the top reason given by HEWs for low utilization (Miller *et al.*, 2013). Lack of awareness of services for newborns, which were relatively new, was especially pronounced (Tefera *et al.*, 2014b, Amare *et al.* 2013, Save the Children, 2013b). Another study in Amhara, Oromia, SNNP and Tigray found that only 4% of caregivers had attended community meetings to discuss child and maternal health issues (Okwaraji *et al.*, 2017).

Awareness regarding the WDA was also low. Only 46% of respondents surveyed in the four agrarian regions had heard of the WDA in their locality (The Last Ten Kilometers Project, 2013). Other studies in Oromia and SNNP in 2014 showed that awareness of WDA was mixed (Save the Children, 2015a,b).

#### Preference for home-based or traditional care

In several qualitative studies in Oromia and SNNP, respondents expressed a preference for home-based care, local drug shops, traditional care, or religious intervention as the first response to childhood illness (Tefera *et al.*, 2014b; Amare *et al.* 2013; Save the Children, 2015a, 2013b, 2015b; Shaw *et al.*, 2016). This preference was especially strong when the illness was perceived as not severe (Shaw *et al.*, 2016, 2017) or when household funds were low (Tefera *et al.*, 2014b; Save the Children, 2013b). This was supported by quantitative household surveys in Oromia and SNNP that also found a preference for informal treatment (Tefera *et al.*, 2014b; Shaw *et al.*, 2015). Distinctions were made between illnesses based on severity and perceptions of aetiology. Traditional medicines were used on common, less severe illnesses, while more specialized or modern treatments were considered more appropriate for severe illnesses (Shaw *et al.*, 2017). Traditional care was preferred because these services were more accessible and caregivers trusted community members more than health workers who were not from the community (Geldsetzer *et al.*, 2014; Shaw *et al.*, 2017).

#### Distance and lack of transportation

Despite the fact that community-based services should reduce distances to healthcare providers, distance remained a major barrier for many people. Distance from health posts and lack of transportation were cited as barriers in several studies. In qualitative research in Amhara, Oromia, SNNP and Tigray, respondents said people from distant communities had a hard time reaching health posts due to long distances and a lack of transportation, including a lack of ambulances (Tefera *et al.*, 2014b; Ethiopia Health and Nutrition Research Institute, 2013; Save the Children, 2015a,b; Shaw *et al.*, 2016; Banteyerga, 2014; Shaw *et al.*, 2017; Kolola *et al.*, 2016; Sibley *et al.*, 2017; Legesse and Dechasa, 2015; Puett and Guerrero, 2015; Tegegne and Mersha, 2017). Furthermore, when HEWs in Oromia were asked what they thought were the common reasons caregivers did not seek care from them, 23% reported that distance was a problem (Miller *et al.*, 2013). Quantitative household surveys from SNNP showed distance and lack of transportation to be important barriers (Tefera *et al.*, 2014a; Ethiopia Health and Nutrition Research Institute, 2013), and a survey in Oromia showed that distance from the nearest health post was significantly associated with use of health post services (Shaw *et al.*, 2015). Another study in Amhara, SNNP and Tigray found that distance to the health post and not having a road for vehicular access to the health post were associated with the utilization of health post services (Ashenafi *et al.*, 2014).

#### Cost of care seeking

Several qualitative studies in Amhara, Oromia, SNNP and Tigray found that cost of care seeking, whether real or perceived, prevented people from remote communities from going to health posts (Tefera *et al.*, 2014b; Ethiopia Health and Nutrition Research Institute, 2013; Save the Children, 2015a; Warren, 2010; Save the Children, 2015b; Shaw *et al.*, 2016; Banteyerga, 2014; Bahir Dar University, 2013; Shaw *et al.*, 2017; Gelaw *et al.*, 2014; Legesse and Dechasa, 2015; Mebratie *et al.*, 2014; Puett and Guerrero, 2015; Tegegne and Mersha, 2017; Jembere *et al.*, 2017). Although services at health posts were free, the cost of transportation to a health centre and fees for services at health centres were important concerns for caregivers (Tefera *et al.*, 2014a). There were also substantial opportunity costs of care seeking, with caregivers needing to spend time on activities such as harvesting and fetching water (Puett and Guerrero, 2015).

#### Need to obtain husband’s permission to seek care

In the 2016 Ethiopia Demographic and Health Survey, one-third of rural mother’s reported that they needed to obtain permission to seek care for their children (Central Statistical Agency [Ethiopia] and ICF International, 2017). In studies in Amhara, Oromia, SNNP and Tigray, mothers listed the need to consult with their husbands to obtain permission to seek care for sick children as an important barrier (Tefera *et al.*, 2014a,b, Amare *et al.* 2013, Ethiopia Health and Nutrition Research Institute, 2013; Save the Children, 2015a, 2013b, 2015b; Bahir Dar University, 2013; Desta *et al.*, 2014; Shaw *et al.*, 2017; Tegegne and Mersha, 2017). The main issue of concern for husbands was the potential expense associated with travel and healthcare, and men were the ones who were decision-makers regarding household expenditures (Tefera *et al.*, 2014a; Save the Children, 2013b). In addition to husbands, grandmothers also had influence on the decision over whether to seek formal care (Amare *et al.* 2013, Degefie *et al.*, 2014).

#### Opposition of traditional or religious leaders

In research in Amhara, Oromia, SNNP and Tigray, respondents reported opposition to formal care seeking by religious and traditional leaders in the community (Tefera *et al.*, 2014b; Save the Children, 2015a; Shaw *et al.*, 2016). In Oromia, religious leaders sometimes discouraged community members from going to the health post because of the family planning services offered there (Shaw *et al.*, 2016).

### Supply-side barriers

#### Health post closure

An important barrier to the utilization of health post services was the inconsistent availability of services. In qualitative research, community members in Oromia and SNNP complained about the limited opening hours of health posts (closed at night and on weekends) and the frequent absence of HEWs from health posts during working hours (Tefera *et al.*, 2014a; Save the Children, 2013b; Shaw *et al.*, 2016; Save the Children, 2013a; Shaw *et al.*, 2017; Legesse and Dechasa, 2015; Jembere *et al.*, 2017). This was supported by quantitative data as well. In Oromia, 21% of caregivers cited the health post being closed as a reason for not using services (Shaw *et al.*, 2015). Another survey in Amhara, Oromia, SNNP and Tigray found that only 52% of health posts received pregnant women during non-working hours (Ethiopia Health and Nutrition Research Institute, 2013). Even HEWs in Oromia reported that health posts were open only 23 hours per week on average, about half of the hours the health post was supposed to be open. HEWs also cited that the health post was not always open as one of the top reasons for low utilization (Miller *et al.*, 2014).

HEWs were away from the health posts for several reasons. They were often called away for trainings, other government work, or other activities, and sometimes they were away for personal reasons (Doherty *et al.*, 2014; FMOH and HEPCAPS II Project, 2015). HEWs reported that it was difficult for them to provide care during off-hours because they did not have their chart booklet, patient register, diagnostic tools or drugs with them at home (Tefera *et al.*, 2014a). Furthermore, some HEWs did not live in the same community as the health post, and those HEWs were more difficult to contact when care was needed (Save the Children, 2013b).

In addition to the inconsistent availability of services at the health post, HEWs rarely provided clinical care in the community through home visits (Save the Children, 2015a; Miller *et al.*, 2013; Puett and Guerrero, 2015). In fact, data from Amhara, Oromia, SNNP and Tigray show that HEWs spent relatively little time providing clinical care in any setting. HEWs spent only 14–16% of their time providing curative services (FMOH and HEPCAPS II Project, 2015; Mangham-Jefferies *et al.*, 2014). Most of their time was spent on health promotion and disease prevention activities or waiting for patients at the health post (Ethiopia Health and Nutrition Research Institute, 2013; FMOH and HEPCAPS II Project, 2015; Mangham-Jefferies *et al.*, 2014).

#### Drug stockouts

In qualitative and quantitative research, caregivers in Amhara, Oromia, SNNP and Tigray mentioned lack of drugs at the health post as a key reason preventing utilization (Doherty *et al.*, 2014; Tefera *et al.*, 2014a; Ethiopia Health and Nutrition Research Institute, 2013; Shaw *et al.*, 2015, 2016; Save the Children, 2013a; Jembere *et al.*, 2017). In a survey in Oromia, 20% of caregivers said they did not use the health post because they thought drugs were not available (Shaw *et al.*, 2015). Two studies provided quantitative cross-sectional assessments of the availability of essential commodities for iCCM (cotrimoxazole, ORS, zinc, ACT, chloroquine, RUTF and RDT). These surveys showed that 69% (Oromia Region, 2012) and 64% (SNNP Region, 2011) of health posts had all essential commodities for iCCM available on the day of the assessment (Miller *et al.*, 2014; Tefera *et al.*, 2014a). With regard to drugs for CNBC, both amoxicillin and gentamycin (for treatment of very severe disease) were available in 84% of health posts. Two-thirds of health posts had ORS, while only 26% had zinc (Berhanu and Avan, 2017).

#### Disrespectful treatment

In a qualitative study in Oromia, some caregivers reported that they felt disrespected or blamed as bad mothers by HEWs. Disrespectful treatment at health posts led mothers to seek care from elders, herbalists or other traditional sources of care (Shaw *et al.*, 2017).

#### Knowledge and confidence of health workers

Several studies showed deficiencies in HEW knowledge, confidence and performance in managing sick children. The Dagu Baseline survey conducted across four regions in Ethiopia reported that the knowledge of HEWs was inadequate with regard to danger signs, newborn care, postnatal care and management of severe acute malnutrition (Okwaraji *et al.*, 2017). In another study in Amhara, Oromia, SNNP and Tigray, trained HEWs reported a self-perceived lack of skill and confidence in treating children, especially newborns (Jembere *et al.*, 2017). The CBNC midline evaluation showed substantial limitations in HEWs’ knowledge of postnatal care, signs of newborn illnesses and management of newborn illnesses (Berhanu and Avan, 2017).

### Potential solutions to demand-side barriers

#### Community education and demand generation

In the early phase of iCCM scale-up, priority was given to the essential programme components of training, supervision and supplying commodities to HEWs. Given gaps in knowledge regarding the causes of childhood illnesses and the availability of services at health posts, the need for campaigns to educate and mobilize community members to improve care-seeking behaviour was emphasized in several studies (Shaw *et al.*, 2017; Demissie *et al.*, 2014; Kolola *et al.*, 2016; Sibley *et al.*, 2017; Legesse and Dechasa, 2015; Mebratie *et al.*, 2014; Mitiku and Assefa, 2017; Negussie and Girma, 2017; Puett and Guerrero, 2015; Ethiopia Health and Nutrition Research Institute, 2013; Okwaraji *et al.*, 2017; Jembere *et al.*, 2017).

The reviewed literature provided information and suggestions that may be useful in designing a community education and mobilization campaign. Respondents reported that HEWs, WDA members and radio were considered credible sources of information (Save the Children, 2015a,b; Tegegne and Mersha, 2017). Radio was the most commonly accessed form of media (41% nationally), but access to any mass media was low among rural communities (32% of women and 46% of men). Therefore, it may be advisable to transmit information by radio, but this must be complemented with other methods. Only 63% of rural men and 32% of rural women were literate (Central Statistical Agency [Ethiopia] and ICF International, 2017), so methods of communication that require literacy, such as written messages on posters and pamphlets, may not be useful.

Various opportunities for discussing and promoting HEW services were suggested, such as during pregnant woman conferences, vaccination campaigns and other community meetings (Save the Children, 2015a,b). Study respondents also suggested using satisfied customers to promote services (Tefera *et al.*, 2014a). Regardless of the methods, special efforts should be made to target distant communities that do not frequently use the health posts (Tefera *et al.*, 2014a; Shaw, 2013).

HEW home visits were reportedly valuable for increasing awareness and use of services (Save the Children, 2015b), and mothers who received a household visit from an HEW were more likely to seek care (Yitayal *et al.*, 2014). Furthermore, mothers who had received training from HEWs and been designated as ‘model households’ were more likely to seek care from the HEW (Yitayal *et al.*, 2014; Gelaw *et al.*, 2014; Kelbessa *et al.*, 2014). Thus, to the extent possible, using HEWs to explain and promote services directly to households would likely be effective (Legesse and Dechasa, 2015).

An evaluation of the effect of family meetings—using adult learning strategies with discussion, negotiation and role-playing to promote care seeking for maternal and newborn services—carried out in the homes of pregnant women by HEWs, community volunteers and traditional birth attendants in 51 kebeles of Amhara and Oromia found that women who participated in the meetings received significantly more interventions compared to women who did not participate. Additionally, there was a positive and significant dose–response relationship between the number of meetings attended and interventions received (4 percentage points for each additional meeting attended). The effect of meeting attendance was stronger for women who attended meetings with other family members (husbands and mothers-in-laws) (Barry *et al.*, 2014).

Another evaluation assessed the use of mobile videos for community behaviour change on maternal and newborn health practices. The videos were produced for each region’s local context and in the local languages. They compared pregnancy and birth experiences in two fictional families, one that attended family meetings and received appropriate care and another that did not. The videos were shown in communities at school compounds, farmers’ training centres, kebele administration buildings and open spaces. Local teams composed of community volunteers, kebele administrators, HEWs, health centre and woreda health office coaches, and project staff organized and conducted the shows and a follow-up session. The evaluation found that attendance was high and that people who attended the viewing of videos had significantly higher recall of key messages than people who did not attend the viewing. The outcome of use of services/interventions was not measured (Desta *et al.*, 2014).

There seemed to be a divide between the younger and older generations in terms of belief in biomedical causes of illness versus supernatural causes. Primary school-aged girls and younger women had higher knowledge of causes of illness and belief in the need for medical care. This group could represent an effective resource from which to train community mobilizers (Sisay *et al.*, 2014).

#### Facilitation and subsidization of transportation to health posts

To address the barrier of distance to the health post, suggestions included creation and maintenance of foot paths to the health post, paying local youths with motorbikes to transport patients or creating local groups of men who can carry sick people to the health post (Save the Children, 2015b; Shaw, 2013). There were also suggestions to subsidize the cost of transportation and opportunity costs for the poorest people and to train private providers in distant communities in iCCM (Tefera *et al.*, 2014a). A study conducted in Amhara, SNNP, Oromia and Tigray reported a functioning mode of transport (a functioning ambulance or other type of motor vehicle) for referral cases, alleviating the cost and difficulty of transportation (Ethiopia Health and Nutrition Research Institute, 2013).

#### Engaging husbands and other family members

Study respondents highlighted targeting of information at a range of key decision makers in the household and in the community. They suggested that information should be targeted not only at mothers but also at husbands, grandparents and other family members (Shaw, 2013; Shaw *et al.*, 2017).

#### Engaging traditional and religious leaders

Both HEWs and community members highlighted the importance of engaging various community leaders to promote the utilization of HEW services. These community members included traditional community leaders, traditional healers, religious leaders and kebele councils (Tefera *et al.*, 2014a; Ethiopia Health and Nutrition Research Institute, 2013; Save the Children, 2015b; FMOH and HEPCAPS II Project, 2015; Shaw, 2013; Shrestha, 2014; Jembere *et al.*, 2017). Through meetings and ongoing discussion with these leaders, their concerns and misgivings about the HEW services and formal healthcare can be discussed and addressed. Furthermore, coordination with traditional and religious healers can promote multifaceted treatment regimens that allow for traditional/religious practices to be combined with formal medical care (Tefera *et al.*, 2014a; Ethiopia Health and Nutrition Research Institute, 2013; Save the Children, 2015b). This integration of traditional ceremonies may also make medical care more acceptable to community members (Save the Children, 2015a; Banteyerga, 2014). An example in Oromia, where coordination with and training of religious and community leaders led to the adoption of community by-laws promoting assisted delivery in health facilities, demonstrated how engagement of local community leaders can improve acceptance of formal health services (Save the Children, 2015b).

### Potential solutions to supply-side barriers

#### Ensuring availability of services at health posts

Respondents in qualitative studies highlighted the need to ensure consistent availability of HEWs at the health post during opening hours and that emergency services would be available at night and on weekends (Shaw, 2013; Jembere *et al.*, 2017). In special cases when a patient cannot travel to the health post, an HEW could go to the community to provide care in the home (Negussie and Girma, 2017). WDA or community groups could also identify pregnant women and other at-risk people and put them in contact with HEWs (Shrestha, 2014).

#### Strengthening supply chain and supervision

As mentioned above, assessments of availability of commodities have shown relatively high to moderate levels of drug availability (Miller *et al.*, 2014; Tefera *et al.*, 2014a; Berhanu and Avan, 2017). However, these do not necessarily reflect the reality of drug availability over time (Jembere *et al.*, 2017). Therefore, it is difficult to determine the extent to which perceptions of lack of drug availability reflect the reality. The perception of lack of availability of drugs may be at least partly related to the pre-iCCM period when health posts often lacked essential commodities (Miller *et al.*, 2014). Nevertheless, it is critical to continue to focus on this issue to ensure a consistent supply of drugs at health posts at all times (Tefera *et al.*, 2014a). Furthermore, mobilizing the community to demand drugs and empowering them to report stockouts could provide an additional layer of supervision and accountability (Tefera *et al.*, 2014a). Strengthened supervision and clinical mentoring is also needed to improve HEWs’ knowledge and performance in managing sick children and newborns in particular (Berhanu and Avan, 2017).

## Discussion

This review showed that continued low utilization of child and newborn health services provided by HEWs can be attributed to a number of demand- and supply-side factors. On the demand side, lack of awareness among community members of childhood illnesses and of the services provided by HEWs, costs associated with seeking formal care and socio-cultural factors affected care-seeking behaviour. Shortcomings in the delivery of iCCM and CBNC services that affected the availability and quality of services further exacerbated the low utilization of services. These findings highlight two truths regarding scaling up community-based health services for impact. First, it cannot be assumed that the availability of services will automatically lead to high utilization of those services. Alongside service delivery, community mobilization, demand generation and promotion of community ownership in community health programmes are keys to acceptance and utilization of services (Ensor, 2004; Farnsworth *et al.*, 2014). Second, supply-side barriers to the utilization of services can be equally as important as demand-side barriers. Thus, there is a need to ensure sufficient demand generation and community engagement as well as a sustained high level of service delivery (availability and quality of services). Only when these two components are satisfied will a programme have the desired impact.

A systematic review of recognition and care-seeking behaviour for childhood illnesses in developing countries found low levels of care seeking from community health workers (CHWs) (Geldsetzer *et al.*, 2014). The demand- and supply-side barriers to utilization in Ethiopia were consistent across the literature and are consistent with previous research in other sub-Saharan African countries (Sharkey *et al.*, 2014; Bedford and Sharkey, 2014; Druetz *et al.*, 2015; Yansaneh *et al.*, 2016; Baume *et al.*, 2000; Muhumuza *et al.*, 2015; Oliver *et al.*, 2012). The challenge now is to identify which strategies and interventions will be most effective in increasing utilization. Previous research has showed that multifaceted demand generation activities that engaged communities were successful in improving the utilization of iCCM services. Key components of community engagement strategies included engagement of local leaders and community elders, interpersonal dialogue with community members, participatory community selection of CHWs and education on danger signs and appropriate treatment for illnesses (Sharkey *et al.*, 2014; Oliver *et al.*, 2012). These interventions could have a positive impact on the demand-side barriers related to community knowledge of childhood illnesses and awareness of services and on socio-cultural barriers. However, further structural changes would be needed to address cost-related barriers and supply-side barriers.

Although the iCCM programme was scaled-up with strong service provision and HEWs provided relatively high-quality care (Miller *et al.*, 2014), service availability and quality may have declined over time (Daka *et al.*, 2020; Getachew *et al.*, 2019). Drug stockouts, in particular, are a major concern (Miller *et al.*, 2014; Tefera *et al.*, 2014a; Berhanu and Avan, 2017). Stockouts not only impede treatment for the present episode, but also erode trust in the health system and discourage future care seeking (Rutebemberwa *et al.*, 2012). Maintaining supplies of essential commodities must continually remain a top priority for the FMOH and implementing partners. Given that parallel supply chains were used for the roll-out of iCCM and CBNC, sustainability of supplies provided through the government supply chain will be crucial (Semu *et al.*, 2019; Legesse *et al.*, 2019). Furthermore, reliable availability of HEWs at the health post must be ensured. HEWs schedules should be managed in a way that ensures that at least one HEW is always available in the health post during working hours. Ensuring availability of CHWs and ensuring consistent supply of commodities have been shown to be associated with higher levels of care seeking and utilization of CHW services in several studies (Sharkey *et al.*, 2014; Littrell *et al.*, 2013; Yeboah-Antwi *et al.*, 2010; Kalyango *et al.*, 2012a,b, 2013; Mukanga *et al.*, 2012; Rutebemberwa *et al.*, 2012). Given the limitations in availability and quality of HEW services, there is a clear need to improve the support provided to HEWs, including enhanced training, supervision, clinical mentoring and provision of commodities, and to ensure that HEWs are consistently present and accessible in communities.

Additional health system-related challenges include resource gaps, inadequate infrastructure, lack of supportive supervision, absence of a well-established referral system, high turnover of HEWs, lack of career path for HEWs, low pay and lack of referral capacity (Assefa *et al.*, 2019; Beyene *et al.*, 2020). These challenges emphasize the fact that the HEWs cannot be successful without adequate support from the health system. Therefore, the structures that support and supervise HEWs, such as the woreda health offices and the referral health facilities, must also be strengthened.

Many of the utilization challenges are related to the fact that the HEP is not a traditional community health programme that delivers services in every community. Many health posts cover a large geographic area that includes several communities. Therefore, the challenges associated with access to health facilities also apply to the health posts. People from distant communities still face challenges in reaching the health post, and HEWs are not known to many community members. The fact that many community members were not aware that HEWs provided curative services demonstrates the distance between HEWs and some of the communities they serve.

The linkages between HEWs and communities clearly need to be strengthened. Given the large coverage areas and the extensive service packages delivered by HEWs, it is probably not possible for HEWs to ensure consistent availability of services in health posts and to regularly carry out clinical outreach services and health promotion activities in all communities. A division of labour that allows HEWs to focus more on clinical activities while another community-based cadre carries out health promotion, community mobilization and home visits for active case finding could be a practical strategy. The WDA presents a promising platform to provide community-level preventive and promotive services. A 2019 systematic review on the contribution of the WDA to maternal and child health in Ethiopia found that the WDA did contribute to improvements in maternal and child health (Yitbarek *et al.*, 2019). However, our review found mixed results regarding the visibility and capacity of the WDA in communities; many community members did not know their WDA leader and the WDA leaders shared many of the communities’ misconceptions about childhood illnesses and the services provided by HEWs. A recent study showed that WDA leaders’ knowledge of maternal, newborn and child health was low and that some key activities were carried out infrequently (Ashebir *et al.*, 2020). Therefore, either the WDA needs to be strengthened or alternative solutions to bring health services and information closer to communities are needed. Other potential solutions would be to expand the cadre of HEWs so that they can serve smaller populations and increase home visits (while the other HEW remains in the health post) and to further engage existing community structures, such as religious leaders, traditional leaders and teachers, to support the work of HEWs in communities.

The results of an independent evaluation of the OHEP project have been published which show that the utilization of services has remained low, possibly due do the short time period of the intervention, delayed implementation of project activities and other implementation challenges (Berhanu *et al.*, 2020; Okwaraji *et al.*, 2020). It is clear that improving the utilization of HEW services must still be considered a top priority, and it will likely be necessary to implement long-term solutions that are integrated into the health system. High geographic variability in the utilization of services provides an opportunity to apply lessons from areas with higher utilization to those with lower utilization (Defar *et al.*, 2019).

There are several limitations of this study. First, much of the literature included in the review was qualitative, so it may be subject to bias and is not necessarily representative of larger populations. Second, the literature is almost entirely from the four large agrarian regions. Therefore, these findings cannot be applied to the other regions, which are characterized by more pastoralist populations. Further study is needed to examine service delivery and the utilization of HEW services in pastoralist areas. Third, the HEP continues to evolve considerably, and this review does not take into account the changes that have taken place since the literature search. As the programme changes substantially, new research will be needed to understand the changing dynamics and to track service delivery and utilization indicators. Finally, this literature search was conducted in early 2018. Since that time, a large number of studies on the utilization of community-based services have been published. Therefore, this study should be taken as a summary of the evidence up to the implementation of the OHEP project.

## Conclusions

The scale-up of iCCM and CBNC represents an admirable and needed effort to make essential healthcare services more accessible for Ethiopian children. The country has recognized that the major challenge to these efforts is the continued low utilization of HEW services. The major barriers to utilization on both the demand and supply sides have been consistently identified across numerous studies. The key challenges now are to ensure consistent availability of high-quality services and to bridge the remaining gap between health posts and communities.

## Supplementary Material

czab047_Supp

## Data Availability

There are no new data associated with this article.
