# Supplementary material for: Barriers to the utilization of community-based child and newborn health services in Ethiopia: a scoping review
Source: Health Policy Plan. 2021 May 27;36(7):1187–96. doi: 10.1093/heapol/czab047 (PMC8496769; doi:10.1093/heapol/czab047)
Supplement: czab047_Supp [file czab047_supp.zip › Table_of_included_documents.docx]

# Appendix 2: Key information from included documents

| **Lead author** | **Title** | **Year** | **Publishing Organization** | **Type of document** | **Journal** | **Study methods** | **Key findings** |
| --- | --- | --- | --- | --- | --- | --- | --- |
| Amouzou | Effects of the Integrated Community Case Management of Childhood Illness Strategy  on Child Mortality in Ethiopia: A Cluster Randomized Trial | 2016 | Johns Hopkins Bloomberg School of Public Health, ABH Services PLC, Mela Research PLC | Journal article | American Journal of Tropical Medicine and Hygiene | Cluster-randomized trial | “Careseeking from HEWs remained generally low in the evaluation area. At endline, caretakers of slightly less than 10% of children aged 2–59 months with at least one of the three illnesses in the previous 2 weeks sought care from an HEW in intervention areas (9.3%) or comparison areas (7.7%). However, these proportions represent a statistically significant increase from the baseline level (5.1% in intervention and 3.6% in comparison areas). The increases in the proportions occurred equally in both study arms, making the difference in differences statistically insignificant. Similar patterns of increases were observed for all three illnesses.” |
| Ashenafi | Effect of the Health Extension Program and Other Accessibility Factors on Care-Seeking Behaviors for Common Childhood Illnesses in Rural Ethiopia | 2014 | JSI Research & Training Institute, Inc., United Nations Children’s Fund | Journal article | Ethiopian Medical Journal | Secondary analysis of survey data | “Appropriate care-seeking behavior increased significantly for all three syndromes between baseline and follow-up surveys, mostly from increases in seeking care from health posts (Table 2). For ARI, utilization of iCCM services from health posts increased 12 percent-points (2% to 14%); for diarrhea, 16 percent-points (4% to 20%); and for fever, 13 percent-points (2% to 15%). There were no statistically significant changes in care-seeking behavior for childhood illnesses from other appropriate sources. The other appropriate sources were mainly health centers (70%, 80%, and to 76% for ARI, diarrhea, and fever, respectively) and private doctors (24%, 13%, and 21%, respectively).  Other statistically significant accessibility factors associated with higher utilization of iCCM services included living closer to a health facility, not having a health center, and not having a road for vehicular access to the kebele.  Women from the least poor quintile were significantly (p<0.05) more likely to seek care for childhood illnesses which was mainly from other appropriate providers compared to those from the poorest quintile, but care-seeking at health posts (i.e., utilization of iCCM services) was unaffected by wealth quintile.” |
| Berhanu | Community Based Newborn Care: Baseline Survey Summary Report | 2014 | London School of Hygiene and Tropical Medicine | Report | N/A | Cross-sectional survey | “The overall average for the four regions showed that among the expected pregnancies, over half were not identified by the health system, and three quarters did not receive any ANC. Similar to health centres, the service utilisation gap was high for optimal PNC (among expected deliveries). (For four regions averaged: 76% no ANC visits, 94% less than 4 ANC visits, 78% no PNC visits, 91% less than 4 PNC visits).  Fourteen percent of all women had one PPC (post-partum care) visit, 6% had two and 3% said they had three visits. For all three PPC visits, the most common source of care was from a HEW followed by a nurse or a midwife.” |
| Berhanu | Community Based Newborn Care in Ethiopia: Quality of CBNC programme assessment  Midline Evaluation Report | 2017 | London School of Hygiene and Tropical Medicine | Report | N/A | Pre-post cross-sectional surveys | “There was a shortage of HEWs in late implementing areas, although despite this shortage, compared with early implementers, a greater proportion reported being available to provide services on weekends and holidays. On average health posts were open for ive working days, with 15% reporting that they were operational for two to four days a week.  On the day of the survey, 97% of health posts had amoxicillin and 91% had gentamycin. A quarter of health posts had expired oral rehydration solution and half had expired zinc.  Postnatal care (PNC), particularly at health posts, was very low.  In this study, 98% of HEWs (100% in early and 96% in late implementing areas) had received CBNC training. However, a quarter of HEWs had not attended annual Integrated Refresher Training. The assessment of HEWs’ unprompted knowledge (without the use of the chart booklet) showed that they had very good knowledge on nutritional counselling and assessment. Though there were gaps in their knowledge across newborn care and signs for sick newborns, HEWs had good knowledge of management and treatment for newborns with a given disease classification.  The experience of the caregivers at health posts was very positive. Exit-interviews showed that they were satisfied with the care that was provided to them by the HEWs, which potentially reinforces positive health seeking behaviours and sustains the demand for community newborn care services.” |
| Demissie | Assessment of Health Care Seeking Behavior of Caregivers for Common  Childhood Illnesses in Shashogo Woreda, Southern Ethiopia | 2014 | JSI Research & Training Institute, Inc., Pathfinder International, University of Camerino, SNNP Regional Health Bureau, Addis Ababa University | Journal article | Ethiopian Journal of Health Development | Cross-sectional survey | “A considerable number of caregivers did not seek care from health facilities (31%): 7.8% purchased medicine from drug shops; 7.2% taken to religious or traditional healers; 10.0% given home treatment, and no care was sought for 9% of children. The main reasons for preference of treatment other than health facilities were treatment expensiveness (41.1%), lack of money for treatment (33.7%), thought the illness was not serious (12.4%), and distance (3.6%).  In this study, mothers’ marital status, their knowledge about the child illness, sex of the sick child and presentation of main symptoms of the child illnesses were found as predictors of health care seeking behavior for childhood illnesses.” |
| Desta | Use of Mobile Video Show for Community Behavior Change  on Maternal and Newborn Health in Rural Ethiopia | 2014 | JSI Research and Training  Institute, Inc., Emory University | Journal article | Journal of Midwifery & Women’s Health | Qualitative focus group discussions, cross-sectional survey | “The mobile video show reached a total of 28,389 mostly young or adult females in 51 kebeles. At endline, mobile video show attendees (vs. non-attendees) reported significantly (P=<.001) higher rates of recall of key MaNHEP messages about use of health extension workers for pregnancy registration, labor and birth notification, and postnatal care.  Participants acknowledged that communities are not generally aware of pregnancy-related problems or the availability and benefits of facility-based birth services and are not willing to accept medical advice from health extension workers (for example, that pregnant women should reduce their household workload). They affirmed that since, traditionally, male household heads control all decisions around pregnancy, it is difficult for women to seek antenatal care or postnatal care without his support. Participants pointed out that these mores are so strong that women often do not disclose their pregnancies to their husbands during the first trimester.” |
| Doherty | Report on the Summative External Evaluation of the Catalytic Initiative (CI)/ Integrated Health Systems Strengthening (IHSS) Programme in Ethiopia | 2014 | Medical Research Council, University of the Western Cape, Save the Children | Report | N/A | Secondary analysis of survey and routine program data, modeling analysis, costing analysis, document review, qualitative interviews | “Almost all the interviews with the implementing partners and the FMOH raised the issue of poor utilisation of the health services by mothers and caregivers of children, especially those under the age of two months old. The 2013 UNICEF report to DFATD concurs with the view that the service utilization by newborns and sick young infants remains very low. This poor utilisation continued despite the introduction of iCCM, training of HEWs and improved provision of pharmaceutical supplies to HPs. The report states that as this age group is most at risk of morbidity and mortality and contributes to over 40% of <5 mortality, reaching them with lifesaving interventions is critical. Data from the JHU Oromia Survey[3](#_ENREF_3) intervention areas shows that, on average, health posts treated 6, 4, 2 and 1 cases of diarrhoea, pneumonia, malaria and malnutrition in the previous month, respectively. Utilisation was much lower in the comparison areas at 2, 1, 0.3 cases of diarrhoea, malaria and malnutrition, respectively in the previous month. According to routine programme data, the most commonly treated condition by HEWs is diarrhoea (ORS) at 38% of all treatments during the project period, followed by pneumonia (28%), malaria (25%) and malnutrition (9%). Estimates from routine data, based on numbers of trained HEWs and total treatments given, reveal an average of 22 treatments per iCCM trained HEW per year (range 19-26).  Reasons given by interviewees for low caseloads at the HPs included: (1) Health seeking behaviour - sick people in the community do not first think of accessing help at a HP; (2) Stock-outs of drugs and other supplies at HPs; (3) No HEWs at HPs. HEWs are involved in other activities (other developmental work or campaigns); (4) Traditionally infants are kept inside for the first 42 days of their lives; and (5) Health posts were not open all the time.  Representatives from the FMOH were very concerned that community health-seeking behaviour contributed to poor utilisation of services at HP level. They were aware that the authors of the Oromia survey had recommended that care seeking needs improvement and that low utilization of the service is caused by lack of awareness and a lack of access. Throughout the interviews and observations it was clear that the FMOH, with the support of UNICEF and the IPs, have attempted to address these problems through improved replenishment of drugs and other supplies at the HPs, changing the policy regarding the opening of HPs so that they are open all day, consolidation of the HDA programme and using HDA volunteers to encourage people with sick children to seek help at a health post. In addition, the ministry introduced the 1-5 networks, by which HEWs mentor HDAs, and hoped this strategy would assist in ensuring that it would be easier to identify sick children within communities. However, a FMOH director acknowledged that the HDAs were not functioning as hoped in mobilising communities but they were confident that with the care-seeking strategies described above, and the improved supervisory structure initiated by UNICEF, services at a HP level would improve leading to increased health post utilisation.  Besides low utilisation of health posts another concern was that very few cases of pneumonia were treated monthly at health posts. HEWs mentioned that they attend to 3 - 5 children with pneumonia a month, although this number varied depending on the season, and did go up to 8 per month. However, a respondent from the IRC said that there was high treatment coverage at health posts in regions where they work. There was a feeling that numbers of cases of pneumonia treated would increase if the HDA, not just the HEWs, were allowed to treat clients, as the HDA are closer to the community and visit a larger number of households (30-50) than the HEWs.  Within rural areas of Ethiopia, amongst children with fever, suspected pneumonia or diarrhoea, whose caretakers sought care, the proportion seeking care from public and private health facilities increased between 2005 and 2011, whilst the proportion seeking care from community providers (government health post/HEW) stayed fairly similar (Figure 32). In both periods, rural areas of Benishangul-Gumuz (an IHSS programme region) had the highest proportion of children seeking care in the community compared to other regions (10%).  Assessing care-seeking at health post/HEW level across wealth quintiles shows a decline in care-seeking at this level across all wealth quintiles between 2005 and 2011 (Figure 33). Amongst appropriate providers*, care-seeking at levels above health post/HEW level increased, from 74% in 2005 to 85% in 2011 (Figure 34). The timing of the 2011 DHS may preclude any conclusions about care-seeking to HEWs since they were only trained in iCCM in 2011. There are also concerns about whether respondents in national surveys are able to distinguish HEWs from facility-based staff.” |
| Ethiopia Health and Nutrition Research Institute | Baseline Evaluation of Maternal and Newborn Health Care Services in 25 Selected Woredas | 2013 | Ethiopia Health and Nutrition Research Institute, Ethiopian Federal Ministry of Health | Report | N/A | Cross-sectional survey, qualitative interviews and focus group discussions | “Overall, it appears that women in the CIFF project areas of the 4 regions were not sufficiently aware of those signs of Maternal and newborn illnesses that indicate the need for treatment…Newborn danger signs reported by mothers mainly concern a few issues, which include fever (59.5%), poor sucking or feeding (41.6%%), and difficult/fast breathing (24%), diarrhea (42.2%), persistent vomiting (16.3%) and infection (10.5%), the other danger signs were reported by a very small percentage of the women and can be considered virtually absent.” |
| Federal Ministry of Health | Health Extension Workers Time Motion Study Complemented by In-depth Interviews  within Primary Health Care Units in Ethiopia | 2015 | Ethiopian Federal Ministry of Health, Harvard T.H. Chan School of Public  Health, Yale Global Health Leadership Institute, JSI Research & Training Institute, Inc. | Report | N/A | Time motion study, qualitative interviews | “Out of the total observed work time, the percentage of total time spent on various activities were as follows: providing health education or services (12.8%); participating in meetings and giving trainings (9.3%); conducting community mapping and mobilization (0.8%); recordkeeping, reporting, managing family folders (13.2%); managing commodities  and supplies (1.3%); receiving supervision (3.2%); receiving training (1.6%); travel between work activities (15.5%); waiting for clients in the health post (or health center in urban settings) (24.9%); building relationships in the community (13.3%); and other activities that could not be meaningfully categorized (4%). The breakdown of health education and services by service package was as follows: hygiene and environmental sanitation (30.3%), family health service (44.7%), disease prevention and control (12.4%), non-communicable diseases 0.6%), and other (11.9%).” |
| Gebre | Newborn-Care Practices and Health-Seeking Behavior in Rural Eastern Ethiopia: A Community-Based Study | 2018 | International Medical Corps, Public Health Research Consultants, ZAAT Health and Development Research PLC, Wellcome Trust Brighton & Sussex Centre for Global Health Research, Addis Ababa University, Save the children | Journal article | Journal of Tropical Pediatrics | Cross-sectional survey | 6.76% of surveyed caregivers sought care from a CHW. |
| Gelaw | Effect of Residence on Mothers’ Health Care  Seeking Behavior for Common Childhood Illness in  Northwest Ethiopia: A Community Based  Comparative Cross-Sectional Study | 2014 | University of Gondar | Journal article | BMC Research Notes | Cross-sectional survey | “Health care seeking behavior was higher among urban mothers (84.6%) than rural mothers (76.7%). Marital status, completion health extension package, and sex of child were significantly associated with health care seeking behavior of urban mothers. Whereas age of child, age and occupation of mothers, educational level of fathers, wealth quintile, and type of reported illness were significantly associated with rural mothers. Perceived severity of illness was significantly associated with both urban and rural mothers for health care seeking behavior.” |
| Institute for International Programs | Independent Evaluation of the Integrated Community Case Management of Childhood Illnesses in Oromia Region, Ethiopia | 2013 | Johns Hopkins Bloomberg School of Public Health | Report | N/A | Cluster-randomized trial | “In terms of the provider of care, HEWs did not stand out as the main care providers for these illnesses. Although care seeking from HEWs were generally, there was a statistically significant increase of 7 percentage points (from 5% to 12%) in case seeking for pneumonia from HEWs in intervention area compared to a non-statistically significant increase of 5 percentages points (from 4% to 9%) in comparison areas.  Care seeking from HEWs remained generally low in the evaluation area. At endline just below 10% of children under-five with at least one of the three illnesses in the past two weeks sought care from and HEW in intervention (9.2%) or comparison area (7.7%). However these proportions represent a statistically significant doubling of the baseline level (5% in intervention and 3.5% in comparison areas). The increases in the proportions however occurred equally in both study arms, making the difference in the differences non-statistically significant. Similar patterns of increases are observed for each illness. Considering pneumonia, the proportion of sick children for whom care was sought from an HEW increased significantly from 4.9% to 11.5% in intervention (p-value=0.0174) and from 4.3% to 9.6% in comparison areas, although non-significant (p-value=0.1208). Care seeking for fever from an HEW also increased significantly from 3.8% to 8.4% in intervention area (p-value=0.0169) and 2.4% to 6.3% in comparison area (p-value=0.0080). For diarrhea, the proportions went from 6.1 to 9.8 in intervention areas (p-value=0.2074) compared to an increase from 3.3% to 8.5% in comparison areas (p-value=0.0090). When the analysis is restricted to children age 2-59 months, the proportions of sick children who sought care from an HEW by type of illness are not markedly different from those among the entire children of under-five.” |
| Institute for International Programs | Qualitative Assessment of the Factors Limiting Care-Seeking for Diarrhea, Malaria and Pneumonia among Children in Oromia Region, Ethiopia | 2013 | Johns Hopkins Bloomberg School of Public Health | Report | N/A | Qualitative interviews and focus group discussions | “Barriers to care-seeking and specifically barriers to care-seeking at the health post were organized around five categories: financial; access; knowledge and information; socio-cultural and religious; and health facility deterrents. These barriers are interrelated and there was significant overlap in the issues discussed.  Financial barriers were the most common barriers suggested by caregivers in reference to care-seeking outside of their community or why they were unable to get preferred sources of care. Financial barriers were due to two primary, direct costs: costs associated with medicines and costs associated with transportation. Money was rarely available on-hand for mothers in this study except during harvest time. Most caregivers were relatively poor and were farmers. Savings was rarely mentioned. Caregivers often mentioned taking out loans to treat their children or selling off livestock and future yield of crops. These strategies could require significant amounts of time, delaying care-seeking and altering preferred pathways of care. A husband’s control over financial resources was occasionally seen as a barrier to care-seeking. In reference to the health post, several caregivers had the expectation that a visit to the health post would likely lead a costly referral potentially delaying or altering care-seeking.  The distance from the household to a higher-level health facility was a frequently mentioned challenge for caregivers. Access to a cheap and regular source of transport as well as emergency transport was perceived as non-existent for most communities selected. Access barriers were also noted for the health post, particularly relating to caregivers in distant sub-villages (greater than one hour[s walk from the health post). Other challenges related to difficult terrain, carrying children and wet conditions were related to transport. Caregivers who did not utilize the health post also commonly mentioned these challenges.  Despite the low educational attainment of most caregivers and especially for mothers, knowledge about causation, symptoms and prevention strategies for diarrhea and malaria was generally high. However, knowledge of the availability of appropriate treatment for diarrhea and malaria was mixed. Knowledge of pneumonia was very low among caregivers. According to most HEWs and VCHWs, knowledge and information barriers were the most significant barriers to care-seeking and, especially, utilization of the health post in their communities. In particular, there was very low knowledge of pneumonia. There was no Afaan Oromo term for pneumonia and those available terms used often overlapped less serious conditions. Many caregivers who did not utilize the health post were unaware that the health post provided treatments for child illness, especially for pneumonia.  Potential socio-cultural barriers largely revolved around the perception that biomedical treatments and the health post were inappropriate for certain illnesses or illnesses attributed to supernatural causes. This was especially common for children under the age of two months who were seen as vulnerable along with their mothers who had recently delivered. Other than those previously discussed, there did not appear to be additional gender-related barriers. Religious beliefs played a smaller role. Most suggested that ‘God acts through medicines’ and religious belief was not a barrier to care-seeking from most sources. However, some HEWs did note that they came into conflict with influential religious leaders over their provision of family planning services.  Although caregivers did note service-related barriers related to health centers, the large majority focused on those barriers related to the services given at health posts. Perceived barriers largely related to: the absence of medications; the absence of the HEW; and the tendency of HEWs to refer children to higher levels of care. There were relatively common perceptions among those that do not utilize the heath post that HEWs and the health post did not have medicines for child illnesses, did not have the specific medicine for their child’s illness or had inferior medicines compared to health centers. These perceptions were also more common among fathers. The absence of the HEW or the hours of the health post were also frequently mentioned. Three sites had only one active HEW posted. Finally, there was a common perception among some who did not utilize the health post that HEWs only or always refer children to the health center. These caregivers saw the utilization of the health post as an unnecessary step in seeking care for their child. HEWs related these perceptions to past experiences of medicine shortages and when referral was more common, prior to the institution of iCCM.  Local Solutions Identified  Although participants in this study had difficulties in making concrete suggestions for preventing or minimizing the barriers mentioned, some solutions were offered.  Caregivers suggested that in addition to education about child illnesses, the HEW could also provide information on costs expected to better prepare them for referral and additional treatments. Some mothers desired economic empowerment such as through additional income generating opportunities that allowed them to have money on hand. Savings was seen as a potential solution, but few mothers had knowledge or concrete solutions for generating and organizing savings. HEWs saw the experiences of HDA members and network of members as instrumental in meeting these needs.  Caregivers suggested engaging local leaders to ensure better maintenance of footpaths from households to the health post. Many also suggested that the HEW should be mobile and carry medicines during outreach activities in the community. Having medicines on hand and bringing the medicines to mothers was seen as one way to reach mothers of children under the age of two months who would otherwise not seek care. Local youths with bicycles and motorbikes paid a stipend by the government were also seen as one potential source of local transportation to and from the health post.  The suggestion for more frequent contact and messages solely devoted to child illness delivered by the HEWs was a consistent theme noted by a majority of caregivers. Caregivers also suggested that HEWs should target specific sub-villages, signage posted (especially letting community members know the medicines available), use clear messages and work with the government to publicize messages on the radio. Caregivers also noted the need for engaging and educating additional populations such as grandmothers, fathers, village leaders, traditional healers, local shop owners and schoolchildren. For the HEWs several of these tasks, especially those related to education, could be taken on by the HDA.  These same activities for knowledge and information barriers were seen as key for counteracting socio-cultural and religious barriers. One HEW suggested that partnerships could be sought and achieved with traditional healers and elders knowledgable about traditional medicines.  Most suggestions relating to health post deterrents related to ensuring the availability of the HEW at the health post, having a wider range of medicines and services available at the health post. These included actions undertaken by the government to: ensure that there are two HEWs at every health post, limit the absences of HEWs due to trainings, extend the hours of the health post, open the health post on the weekends a limited number of hours and monitor the performance of HEWs.” |
| Kelbessa | Level of Health Extension Service Utilization and  Associated Factors Among Community in Abuna Gindeberet District, West Shoa Zone, Oromia  Regional State, Ethiopia | 2014 | West Shoa Zonal Health Office, Haramaya University | Journal article | BMC Health Services Research | Cross-sectional survey | “The proportion of community utilization of health extension service was 39%. Age (AOR = 2.52; 95% CI = 1.53-4.15), occupation (AOR = 3.79; 95% CI = 1.64-12.5), knowledge of community on health extension service (AOR = 0.25; 95% CI = 0.18-0.36), community participation in planning of health extension activities (AOR = 0.22; 95% CI = 0.15-0.33) and graduation of model family (AOR = 0.74; 95% CI = 0.47-0.76) have statistically significant association with community health extension services utilization.” |
| Kolola | Health Care Seeking Behavior for Common  Childhood Illnesses in Jeldu District, Oromia  Regional State, Ethiopia | 2016 | Debre Berhan University, Addis Ababa University | Journal article | PLOS One | Cross-sectional survey | “Health care seeking behavior for childhood illnesses was delayed and decision to seek  care from health facilities was influenced by worsening of the illnesses. Thus, community level promotion of prompt health care seeking is essential to enhance the health care seeking behavior for childhood illnesses in the locality.” |
| Last Ten Kilometers Project | Integrated Community Case Management (iCCM) Survey in Amhara, SNNP, and Tigray Regions | 2013 | Last 10 Kilometers Project | Report | N/A | Cross-sectional survey | “The study found that adoption of iCCM within the health extension program at scale substantially increased the care-seeking behaviors for children sick with acute respiratory infection, diarrhea and fever. The improvement in care seeking behaviors for common childhood illnesses were mainly contributed by the increase in utilization of iCCM services from health posts. The major barriers to obtaining iCCM services were: the mother thought that the child will get better (42%), domiciliary responsibilities of the mother (25%), financial reasons (16%), and not a custom to seek care (10%). Significant fraction of the mothers (39%) were also unaware regarding the availability of iCCM services at the health post.” |
| Last Ten Kilometers Project | Trends in reproductive, maternal, newborn and child health care practices in 115 L10K woredas: Analyses of three rounds of survey data | 2015 | Last 10 Kilometers Project | Report | N/A | Cross-sectional surveys | “There were no improvements in care‐seeking behaviors for ARI, fever and diarrhea between Round I and Round II; however, improvement in the care‐seeking behaviors were observed between Round II and Round III. A sharp increase in the treatment practice from any provider for ARI from Round II (around 35 percent) to Round III (69 percent) was observed. Similarly 66 percent reported to have sought treatment from a provider for fever during Round III—and increase from 41 percent in Round II. About 36‐38 percent of the respondents mentioned receiving treatment from HCs for fever and ARI, and only 17 percent mentioned taking their children to HPs. Similar trend has been observed regionally. In Round III, 52 percent of children aged 0‐23 months with ARI received antibiotics, while only 3 percent received any anti‐malarial drugs for fever.  Fifty‐one percent of children of 0‐23 months of age with diarrhea were taken to health providers during Round III. Twenty‐nine percent sought treatment from a HC and 15 percent from a HP. Consequently, 34 percent of children with diarrhea received oral rehydration solution in Round III, a modest increase from 28 percent in Round II. The practice of giving zinc for diarrhea has also increased from 3 percent in Round II to 13 percent in Round III. The improvement in care‐seeking behaviors for ARI, diarrhea and fever can be attributable to the iCCM strategy the HEP adopted in 2010 and expanded in 2011.” |
| Legesse | An Assessment of Child Immunization Coverage  and Its Determinants in Sinana District, Southeast  Ethiopia | 2015 | Wollega University | Journal article | BMC Pediatrics | Cross-sectional survey | “Almost all, 584(98.8%) of respondents reported that they have access to health facilities that provide immunization services. Majority of them, 537(92.0%) reported that they have more access to health post, 382(76.9%) have access to services provided during outreach and 270(46.2%) have access to health center.  From the total, 573(97.9%) of the households were visited by HEWs and 564(96.4%) were given information on immunization by HEWs regularly. Of the total 576 mothers visited health facilities for vaccination, 119(20.7%) turned back home without getting their children vaccinated. From this, the 69(58.0%) were due to the unavailability of the service providers and 62(52.1%) were due to lack of vaccine at these facilities.” |
| Mebratie | Healthcare-Seeking Behaviour in Rural Ethiopia: Evidence From Clinical Vignettes | 2014 | Erasmus University Rotterdam, Ethiopian Economic  Association, Addis Ababa University, Georgetown University | Journal article | BMJ Open | Cross-sectional survey | “In the case of malaria, households in the richer quintiles are 2.1 (95% CI 0.89 to 5.08, p=0.09) to 3.4 times (95% CI 1.37 to 8.35, p=0.01) more likely to seek modern care as compared with those in the poorest quintile.  Household heads with informal education are 1.6 times (95% CI 1.07 to 2.46, p=0.02) more likely to take their children to health centres for ARI/pneumonia (baseline is health posts) which potentially offer a higher quality of care as compared with household heads with no education. Education does not exert much of an influence on care-seeking behaviour for diarrhoea. However, in both cases, there is clearer evidence that richer households are more likely to access hospitals as opposed to health posts.” |
| Miller | Assessment of ICCM Implementation Strength and Quality of Care in Oromia, Ethiopia | 2013 | Johns Hopkins Bloomberg School of Public Health | Report | N/A | Cross-sectional survey | “Utilization of iCCM services is low. Intervention health posts had an average of 16 sick child consultations per health post in the previous month. Nearly all of these children were between 2-59 months of age, with virtually no children under two months seen in the previous month. There was large variation in utilization among health posts, with a range of consultations per month from zero to 95.  Although utilization in intervention areas was low, it was over three times higher than in comparison health posts, which saw an average of only five children per health post in the previous month. The number of children seen ranged from zero to 32.  During the HEW interviews, data collectors asked HEWs what they thought were the main reasons caretakers do not bring their sick children to the health post for care. The most common responses among HEWs in intervention health posts were:   - Caretakers are not aware of the availability of case management services in the health posts (30%), - Distance from home to health post (23%) - Caretakers want injectable drugs (13%)   Utilization is strongly positively associated with availability of iCCM commodities, supplies, and job aids, with an increase of 1.9 children seen per month for every one unit increase in the index of commodity, supply, and job aid availability (with a total of 15 items). In other words, a health post with all 15 items available saw on average 19 more children in the month than did a health post with only five items available.  Health posts that received supervision on iCCM in the previous three months had a higher average number of consultations and those that received no supervision and health posts that received supervision that included clinical reinforcement (register review or observation of case management) had even higher utilization. Health posts that received no supervision saw an average 8.9 children in the previous month, compared to 15 for health posts that received supervision without clinical reinforcement and 17.6 for health posts that received supervision with clinical reinforcement.  Finally, health posts that were open and offering clinical services for more hours in the previous week had a higher average number of consultations. The average number of consultations increased by 0.24 for every hour increase in the time the health post was open. Therefore, a health post that was open 40 hours in the previous week saw 7.2 more children than did a health posts that was open for 10 hours.  According to the HEWs, intervention health posts were open and offering clinical services an average of 23 hours in the previous week. HEWs also reported that in the previous day, they spent about four hours providing or offering clinical services in the health post. They spent only half an hour providing clinical services in the community and nearly one hour carrying out community mobilization/education activities. In total, they reported spending an average of about six hours on work-related activities in the previous day. HEWs in comparison health posts reported that the health post was open about the same number of hours in the previous week (20 hours) as in intervention health posts. HEWs reported spending only two hours in the previous day providing/offering clinical services in the health post. They spent more time (1.5 hours) doing community mobilization/education in the community. HEWs in the comparison areas reported spending a total of 5.5 hours on work-related activities in the previous day.” |
| Miller | Integrated Community Case Management of Childhood Illness in Ethiopia:  Implementation Strength and Quality of Care | 2014 | Johns Hopkins Bloomberg School of Public Health, ABH Services PLC, United Nations Children’s Fund, Federal University of Pelotas | Journal article | American Journal of Tropical Medicine and Hygiene | Cross-sectional survey | “Intervention health posts saw an average of 16 (95% CI = 13–19) sick children in the previous 1 month (range = 0–95) compared with 5 (95% CI = 2–8) sick children seen in comparison health posts (range = 0–32). Virtually no children under 2 months of age were seen in intervention or comparison areas.  Intervention and comparison health posts were reportedly open and offering clinical services for an average of about 20 hours in the previous week.” |
| Mitiku | Caregivers’ Perception of Malaria and Treatment‑Seeking Behaviour for Under Five children in Mandura District, West Ethiopia: A Cross‑Sectional Study | 2017 | Wollo University | Journal article | Malaria Journal | Cross-sectional survey | “Of those children brought to treatment, 129 (74.6%) received treatment in the public health institution and 44 (25.4%) received treatment from local private providers like drug vendors. Some of the children (18.5%) received treatment at home before they were taken to health facility.  Overall, 56.2% of caregivers had low perception about susceptibility to malaria infection and 51.1% had low perception about severity of the disease. Nearly 63.7% of caregivers had low perceived barrier to seek treatment for under-five children. However, 51.3% of caregivers had low perception of the benefits of seeking treatment. More than 60% of respondents had low cue to action and 58.7% had low self-efficacy.” |
| Negussie | Is the Role of Health Extension Workers in the Delivery of Maternal and Child Health  Care Services a Significant Attribute? The Case of Dale District, Southern Ethiopia | 2017 | Yirgalem Hospital Medical College | Journal article | BMC Health Services Research | Cross-sectional survey | “Mother’s educational status, listening to the radio, information delivery by HEWs about MCH services and home visits in the last 6 months by HEWs were significant in the bivariate analysis with p-value ≤0.05. In order to identify predictors of good MCH service utilization, the possible confounding effect was controlled using multiple logistic regression analysis; and found that mothers who listen to the radio (AOR 4.62; CI 1.66–12.85) and who had received information about MCH services by HEWs (AOR 2.09; CI 1.06–4.14) were more likely to use MCH services than mothers who had not.  Out of the mothers who knew MCH services are given in Health Posts, the following proportion of mothers knew that the services are available in HPs; 56.8% - family planning, 51.5% - ANC, 44.4% - delivery, 45.6% - PNC, 80.5% - immunization, 60.2% - growth monitoring and 45.5% - sick baby care services. Moreover, 195 (74.4%) of the mothers also described that HEWs were available at HPs during their last visit for MCH services.” |
| Noordam | Care Seeking Behaviour for Children with  Suspected Pneumonia in Countries in Sub-  Saharan Africa with High Pneumonia  Mortality | 2015 | Maastricht University, United Nations Children’s Fund | Journal article | PLOS One | Secondary analysis of survey data | “In Ethiopia the percentage seeking care from an ‘appropriate’ provider in rural areas is 25, in contrast to 47% in urban areas.  This significant association between younger age of the child and care seeking from ‘appropriate’ providers was also found for Ethiopia and DRC. In general, the influence of a caregiver’s education, when controlling for other variables shown in Table 3 shows that higher educated caregivers and higher wealth quintiles were also associated with higher levels of care seeking from ‘appropriate’ providers in this group of countries.  In Tanzania, Ethiopia, Nigeria and Burkina Faso, caregivers from the richest quintile were much more likely as those from the poorest quintile to seek care for their child with suspected pneumonia, after controlling for age and sex of the child, urban-rural residence and education, with odds ratios ranging from 4.7 (95% CI 1.5– 15.1) to 9.4 (95% CI2.3– 39.3).” |
| Okwaraji | Community-Based Child Care: Household and Health-Facility Perspectives | 2017 | London School of Hygiene and Tropical Medicine | Report | N/A | Cross-sectional survey | “For each child between the age of 2–59 months, caregivers or mothers were asked if the child had experienced symptoms of illness including diarrhoea; a cough accompanied by fast breathing or difficulty breathing; or a fever in the two weeks preceding the survey. Caregivers were also asked if care was sought when the child was ill. Among 2,873 children between the age of 2–59 months residing in the study area, 6% (N=166) reportedly had an illness episode during the two weeks preceding the survey. Of these, 55% (95% CI 47–63) had sought care from a formal health provider (health post, health centre, hospital or private clinic).  Among 2,873 children residing in the study area, 5% (N=139) reported to have suspected pneumonia, diarrhea or fever during the two weeks preceding the survey. Of these, 47% (95% CI 39–56) received antibiotics, ORS, zinc or antimalarial treatment.  Figure 10 shows the place care was sought for children who reported to be sick in the two weeks prior to survey. The most common place was from a health centre (41%), with 24% seeking care from a health post and 25% from a private clinic.  The caregivers’ knowledge of signs of illness in the newborn baby is shown in Figure 11. Seventy-four per cent of caregivers had unprompted knowledge of at least two or more danger signs of newborn illnesses. Out of eleven common signs or symptoms linked to newborn illnesses, the caregivers were most aware of reduced feeding, diarrhoea, unusually hot or cold baby, and difficult or fast breathing.  Among 2,873 children between the age of 2–59 months residing in the study area, 6% (N=166) reportedly had an illness episode during the two weeks preceding the survey. Of these, 55% (95% CI 47–63) had sought care from a formal health provider (health post, health centre, hospital or private clinic). The most common illnesses during two weeks before the survey for children below the age of 2–59 months were fever, diarrhoea and suspected pneumonia. Among the 2,873 children, 5% (N=139) reported to have suspected pneumonia, diarrhoea or fever during the two weeks preceding the survey. Of these, 47% (95% CI 39–56) received antibiotics, ORS, zinc or antimalarial treatment.” |
| Puett | Barriers to Access for Severe Acute Malnutrition Treatment Services in Pakistan and Ethiopia: A Comparative Qualitative Analysis | 2014 | Action Against Hunger | Journal article | Public Health Nutrition | Qualitative interviews and focus group discussions | “Common barriers were related to distance, high opportunity costs, knowledge of services, knowledge of malnutrition and child’s refusal of ready-to-use foods. While community sensitization mechanisms were generally strong in these well-performing programmes, in remote areas with less programme exposure, beneficiaries experienced barriers to remaining in the programme until their children recovered.” |
| Save the Children | Factors in the Operating Environment  Influencing Community-Based Newborn Care | Not specified | Save the Children | Report | N/A | Qualitative interviews and focus group discussions | “The availability of free treatment was cited by many respondents as a clear factor in facilitating care-seeking in the community, though there were some factors that hampered access. Most community members indicated that opening hours of health posts are short and somewhat unpredictable, particularly when both HEWs assigned to a kebele are engaged in outreach or other community activities. Health posts are generally closed on weekends, and this can create potential problems as newborns that become ill require treatment through the weekend. There were several accounts, however, of HEWs working on weekends to provide treatment and support when needed.  Medicine stock outs are another factor that impacts community use of the health post. If families seek care and the health post is closed or medicines are not available, this discourages care-seeking. If one medicine is unavailable at the health post, there is a tendency to assume this extends to all drugs. Conversely, the knowledge that medicines are in stock, or accounts of successful treatment, tends to spread rapidly by word of mouth and may facilitate utilization.  Reports of home visit coverage by HEWs and volunteers were variable. A few mothers said they had not been visited by HEWs, while others said the HEWs made visits frequently, were always available to the community, or would visit even when they were busy. Generally, HEWs were perceived as more available if they lived in the kebele where they worked. Most communities were familiar with volunteers, and they were perceived to make home visits more frequently than HEWs. Households far from the village center or in isolated areas tended to have fewer pregnancy and PNC visits by volunteers and HEWs than those residing more centrally.  HEWs and volunteers noted several common barriers in the operating environment that influenced home visit coverage. Walking alone is often a safety concern. Distance poses a problem for some, while for others challenges arise from poor roads and difficult terrain, particularly during the rainy season. During the agricultural season, volunteers and pregnant women are also very busy with livelihood responsibilities. Holidays, market days and funerals also lead to delays in visits. Seasonal migration and some women returning to their mother’s village to give birth sometimes impedes follow-up.” |
| Save the Children | Factors Influencing Care and Treatment for Newborn Illness | Not specified | Save the Children | Report | N/A | Qualitative interviews and focus group discussions | “Cultural practices around seclusion of mothers and newborns after birth pose barriers to assessing for danger signs during home visits. Seclusion of the newborn is influenced by traditional beliefs that newborns require protection from cold, wind and direct sunlight.  In East Shoa, it is customary for the mother and newborn to live in seclusion until the baby has been blessed and named by a spiritual leader through the ritual known as amechisa. This practice can take place anywhere from 40 to 80 days following birth. During this time, neither mother nor newborn can travel outside of the home, and only the mother and those present at the time of delivery can touch the baby. In households where amechisa is practiced, some volunteers observe the baby while being held by the mother. HEWs noted that following discussions with community and spiritual leaders, most families accept assessment of the newborn.  In Sidama, fear of the evil eye or a malignant spirit - known as budda - leads families to isolate the newborn from others. Volunteers have made some inroads by lightly spitting when assessing the baby for danger signs, as it is believed that budda cannot spit. HEWs may be allowed to assess newborns more readily than volunteers due to their professional standing.  Newborn illness and care-seeking are strongly influenced by local beliefs. Knowledge of danger signs is limited, and concepts of specific newborn illnesses in some communities call for traditional and sometimes harmful remedies. Underpinning the continued use of traditional remedies is a common belief that the newborn is too small or too fragile for medicines obtained from health facilities. At the same time, many community members believe several traditional practices are in decline. Furthermore, some community members believe seclusion practices would not prevent them from seeking care at a health facility if it became necessary.  In Sidama, hamessa, a liquid made from the bark and leaves of different trees, refers both to an illness and a cure. It is given to the newborn shortly after birth or as treatment when the baby falls ill. Many in the community thought this practice was in decline, particularly fathers and grandmothers, though grandmothers were reluctant to discuss it in detail. Some mothers were unhappy that they had been counseled against using hamessa.  Both fathers and mothers play a role in the decision to seek medical care, though the father is solely responsible for gathering money for transport and treatment, which can introduce delays to reaching care. Awareness of newborn services at health centers in both study arms and at health posts in the community-based PSBI treatment arm is generally limited, though most respondents noted they would like services at the health post. Reasons for this include the closer proximity of the health post compared to the health center and familiarity with community HEWs.  A few fathers expressed concern about the type of medicine that would be given to their baby. Fear that newborns were too small to take medicines was common.  Though some mothers indicated they received a visit from an HEW shortly after birth, several volunteers expressed that the HEWs were not routinely available at the health post, and thus could not be notified. HEWs that lived in town and not the village were more difficult to contact following birth. HEWs indicated they made visits as soon as they heard of a birth, while acknowledging that competing activities pose a challenge to early PNC visits.” |
| Save the Children | The Community-Based Interventions for Newborns in Ethiopia Study | Not specified | Save the Children | Report | N/A | Cluster-randomized trial | “In a before and after evaluation, COMBINE’s package of pregnancy and PNC home visits by HEWs and volunteers was successful in achieving high levels of home visit coverage, increased coverage of several important MNH practices, and reduced overall neonatal mortality. The package included counseling on MNH and danger signs during pregnancy/delivery, assessment of the mother and newborn for danger signs, and referral of newborns with PSBI to health centers for treatment.  As summarized in Figure 2, among women who delivered less than 60 days preceding the COMBINE endline census, 40% received a pregnancy home visit (38% from a volunteer and 9% from an HEW), 19% received a PNC visit within 2 days (14% from a volunteer and 6% from an HEW), and 33% received a PNC home visit within one week of birth (23% from a volunteer and 13% from an HEW).  From baseline to endline, substantial increases were observed in several important care-seeking and newborn care practices. More women sought ANC and more women delivered their baby with a skilled birth attendant. In addition, mothers were more likely to feed their newborn colostrum (first milk), engage in exclusive breastfeeding, and avoid the potentially harmful practice of bathing their babies immediately after birth (which can contribute to heat loss and hypothermia).  COMBINE’s package of pregnancy and PNC home visits by HEWs and volunteers, in combination with community-based treatment of PSBI by HEWs when referral to health centers was not possible or accepted, increased care-seeking and treatment, with a substantial reduction in post day 1 neonatal mortality observed.  There was a large increase in the number of newborns with PSBI seen at facilities over the course of the study, particularly from the beginning of 2012. Most of this increase was at health posts in the community-based treatment arm, though there were also modest increases at health centers in both study arms. There was also a large increase in the number of newborns initiating treatment for PSBI, again particularly from the beginning of 2012, and mostly at health posts in the community-based treatment arm.” |
| Save the Children | Saving Children’s and Mothers’ Lives in Remote Konso and Derashi Districts in Ethiopia’s Southern Nations, Nationalities, and People’s Region: Final Evaluation | 2013 | Save the Children | Report | N/A | Document review, observation, qualitative interviews | “Postnatal care also increased for both the mother and baby: by 17% (26.1% to 43.1%) in Derashie and 29% (30.8% to 59%) in Konso, and for the baby by 31.5% (21.8% to 53.3%) in Derashie and by 28.2% (26.3% to 54.5%) for the mother.  Care seeking for children who had fever increased in both districts: by 19.9% (55.5% to 71.4%) in Derashie; and by 20.7% (56.4% to 77.1%) in Konso. Use of ORS when a child had diarrhea increased in both districts: by 42.8% (27.7% to 70.5%) in Derashie; and by 36.8% (29.7% to 66.5%) in Konso. However, water treatment was poor in both districts at both baseline and endline. Care seeking for children with cough was already good in both districts at baseline and increased slightly in each. In Derashie, care seeking increased by 18.5% (64.5% to 84%); while the increase in Konso was only 9.2% (67.5% to 76.7%).  On the supply side health posts and health centres had CCM and IMNCI supplies, drugs and equipment. On the demand side, interviews triangulated through varied respondents confirmed active promotion of preventive family care practices, care seeking, and referral. Health posts are increasingly becoming significant primary providers of newborn, child and maternal care providers. However, preliminary findings from an HMIS review show still low CCM and IMNCI utilization although it is improving.” |
| Save the Children | Rapid Assessment on Barriers to Early Pregnancy Identification, Focused Antenatal Care, Skilled Birth Attendance and Postnatal Care Service Utilization in East Shewa Zone, Oromia Region | 2015 | Save the Children | Report | N/A | Qualitative interviews and focus group discussions | “Regarding post natal care services, HEWs were reported to be the key actors who influence women’s attendance of PNC services. One of the barriers for utilization of PNC services was the deep rooted tradition which discourages mothers from going out of home for two months after delivery. Post natal care home visits are also carried out by the HEWs and mid wives when available. However, the service is not consistent and comprehensive because of limited number of health workers compared to the demand of services. The most credible sources of information about health issues are radio and health workers. However, face to face interaction was reported to be the most effective methods to deliver information.  In order to address the main barriers related to pregnancy identification and notification, targeted and focused SBCC activities need to be implemented to raise awareness on major signs of pregnancy with specific focus on menstrual cycle and morning sickness. In addition, benefit of utilizing ANC, health facility delivery and PNC services should also be focused in social and behavior change communication efforts. Furthermore, trainings should be provided for health workers to pay more attention to early pregnancy identification as this is the entry point for the key services in the MNCH continuum of care. Existing initiatives that include the registration of pregnant women, monthly pregnant women conferences, engagement of religious and community leaders need to be strengthened further. High level advocacy activities are also required to strengthen the human resource for community outreach services for PNC service and to promote availability of return ambulance services to increase utilization of PNC and health facility delivery services, respectively.” |
| Save the Children | Rapid Assessment of Determinants, Factors and Opportunities to Early Pregnancy Identification, Focused Antenatal Care, Skilled Birth Attendance and Postnatal Care Service Utilization in Gurage and Sidama Zones of SNNPR | 2015 | Save the Children | Report | N/A | Qualitative interviews and focus group discussions | Postnatal care service utilization was found to be far less than expected in both zones. The main reasons were the longstanding assumption in the community that there is nothing to worry about once a mother gave birth safely and fear of being asked to pay for the service. In addition to this, traditional beliefs and practices, lack of transportation, long distance from home to health facility and the limited number of HEWs and midwifes who are supposed to provide home based PNC service were reported as barriers for PNC service utilization. The most common health problems reported among newborns were breathing difficulty, diarrhea , refusal to breastfeed, stomach aches,fever, Jaundice, skin rush and diarhea. Perceived/ fear of financial costs of treatment and long rooted traditional ways of treating newborns were reported to be the main barriers for newborn health seeking behavior in both Gurage and Sidama zones. Especially in Sidama zone, “Amessa” is the most common traditional herbal treatment given to newborns when they are sick. However, the practice was reported to be decreasing from time to time. |
| Shaw | Determinants of Utilization of Health Extension Workers in the Context of Scale-Up  of Integrated Community Case Management of Childhood Illnesses in Ethiopia | 2015 | Johns Hopkins Bloomberg School of Public Health, ABH Services PLC | Journal article | American Journal of Tropical Medicine and Hygiene | Cross-sectional survey | “We found low utilization of HEWs with only 9.3% of caregivers of a child sick with diarrhea, fever, and/or pneumonia in the previous 2 weeks taking their child to HEWs in both iCCM and routine areas. There was a higher likelihood of utilization of HEWs in iCCM areas (OR: 1.44; 95% CI: 0.97–2.12; P = 0.07), but this effect disappeared after accounting for confounders. In iCCM areas, maternal education, illness type, and distance were associated with utilization. Perceptions of illness severity and service quality were the primary reasons given for not utilizing the health post.” |
| Shaw | Access to Integrated Community Case  Management of Childhood Illnesses Services in  Rural Ethiopia: A Qualitative Study of the  Perspectives and Experiences of Caregivers | 2016 | Johns Hopkins Bloomberg School of Public Health, ABH Services PLC | Journal article | Health Policy and Planning | Qualitative interviews and focus group discussions | “In spite of the HEW being a core component of iCCM, we found that the lack of availability of HEWs at the health post was one of the most common barriers to the utilization of iCCM services mentioned by caregivers. Financial and geographic challenges continue to influence caregiver decisions despite extension of free child health services in communities. Acceptability of HEWs was often low due to a perceived lack of sensitivity of HEWs and concerns about medicines given at the health post. Social networks acted both to facilitate and hinder use of HEWs. Many mothers stated a preference for using the health post, but some were unable to do so due to objections or alternative care-seeking preferences of gatekeepers, often mothers-in-law and husbands.” |
| Shaw | A qualitative exploration of care-seeking pathways for sick children in the rural Oromia region of Ethiopia | 2017 | Johns Hopkins Bloomberg School of Public Health | Journal article | BMC Health Services Research | Qualitative interviews and focus group discussions | “Caregivers often described the act of care-seeking for a sick child as a time of considerable uncertainty. In particular, mothers of sick children described the cultural, social and community-based resources available to minimize this uncertainty as well as constraints and strategies for accessing these resources in order to receive treatment for a sick child. The level of trust and familiarity were the most common dynamics noted as influencing care-seeking strategies; trust in biomedical and government providers was often low.” |
| Sibley | Improving Maternal and Newborn Health Care Delivery  in Rural Amhara and Oromiya Regions of Ethiopia Through  the Maternal and Newborn Health in Ethiopia Partnership | 2014 | Emory University, University Research Company,  LLC, JSI Research & Training Institute, Inc. | Journal article | Journal of Midwifery and Women’s Health | Baseline and endline cross-sectional surveys | “The mean proportion of the CMNH care package elements that women reported were received from a health extension worker, community health development agent, or TBA at their most recent birth increased from baseline to endline (Amhara 18%-70% and Oromiya 43%- 87%, P=<.001 for both).  There were significant increases from baseline to endline in the proportion of women who reported that they knew the health extension workers, community health development agents, and TBAs in their own kebele. At endline, most women knew the health extension workers (91%) and TBAs (92%) in their kebele.  With one exception (Amhara women’s level of trust in TBAs to provide pregnancy care), women’s self-assessed level of trust in health extension workers, community health development agents, and TBAs to provide the components of CMNH care increased significantly from baseline to endline. At endline, the mean self-ratings for trust in health extension workers, community health development agents, and TBAs to provide the combined care components were 4.2, 3.9, and 3.8, respectively (1 = least trust, 5 = most trust).  There were large, significant increases in the proportion of women and newborns who received any postnatal care from baseline to endline in both regions. In the Amhara and Oromiya regions, respectively, the use of skilled providers did not change significantly, but the use of health extension workers increased substantially (46%-70% and 31%- 59%, respectively). At endline, 84% of Amhara and 77% of Oromiya women used a skilled provider or health extension worker for postnatal care.” |
| Sibley | Appropriateness and Timeliness of Care-Seeking for Complications of Pregnancy and Childbirth in Rural Ethiopia: A Case Study of the Maternal and Newborn Health in Ethiopia Partnership | 2017 | Emory University, for Social  Development, Amhara Regional Health Bureau, Oromia Regional Health Bureau, Harvard Medical School | Journal article | Journal of Health, Population and Nutrition | Secondary analysis of survey and qualitative data | “…Factors impeding care-seeking from a biomedical provider included rain, nighttime, distance, lack of transportation or money, the mother’s condition (e.g., too weak and/or unclean and traditional postpartum restrictions on movement) as well as the baby’s condition (e.g., too small or too young, not baptized, and the illness requires a local treatment). Factors impeding care-seeking were similar to those mentioned by the families of babies who survived. In addition, they mentioned difficulty calling for an ambulance, losing hope, belief that the baby would get better, and fear of evil eye.  Factors facilitating care-seeking included advice of health extension workers, the ability of health extension workers to refer, free care, and fear of traditional care,…and proximity of the health facility.” |
| Tadesse | Utilization of Integrated Community Case Management Services in Three Regions in Ethiopia After Two Years of Implementation | 2014 | Save the Children, Le Monde Health and Development Consultancy | Journal article | Ethiopian Medical Journal | Analysis of routine program data | “Treatment rates (per 1000 children per year) were low for all diseases in Ethiopia fiscal year (EFY) 2004 (July 2011 to June 2102): 11.9 for malaria (in malarious kebeles only), 20.3 for severe acute malnutrition (SAM), 21.2 for pneumonia, and 29.2 for diarrhea, with wide regional variations, except for pneumonia.  During the same time period, 64% of HP treated five cases or less, and 24% saw between 6-10 cases per month. Only 2% (12/622) of HPs saw more than 15 cases monthly, 11 of which were in SNNPR.  HEWs saw 60% more sick children 2-59 months of age in the first quarter of 2005 EFY as compared to same period in 2004 (3rd quarter 2012 and 2011, respectively (Figure 5). Similarly, HEWs saw 73% more sick young infants during the same period.” |
| Tefera | Factors Influencing the Low Utilization of Curative Child Health Services in Shebedino District, Sidama Zone, Ethiopia | 2014 | Save the Children | Journal article | Ethiopian Medical Journal | Cross-sectional survey, qualitative interviews and focus group discussions, inventory of drugs and supplies | “The many barriers to use of evidence-based treatment included: (1) home remedies of uncertain effect and safety that delay care-seeking; (2) absent decision-maker; (3) fear of stigma; (4) expectation of non-availability of service or medicine; (5) geographic and financial barriers; (6) perception of (or actual) poor quality of care; and (7) accessible, available, affordable, reliable, non-standard, alternative sources of care.” |
| Tefera | Illness Recognition, Home Care, and Care-Seeking for Sick Infants Less Than Two Months of Age in Shebedino District, Sidama Zone, Ethiopia | 2014 | Save the Children | Journal article | Ethiopian Medical Journal | Qualitative focus group discussions | “Reported decision-making about care for young infants was widespread and included parents, any literate or educated person in the household, neighbors, and grandmothers. If the mother was alone, she could decide to seek care outside the home after consulting elders because “[she] has no power to see when her child is dying” and because “[she] cannot keep quiet while our children are dying.”  Barriers to seeking health post care were: (a) lack of knowledge that curative services were available for young infants; (b) fear of evil eye; (c) social stigma (“People will say, ‘She gave birth yesterday and her infant is sick today!’”); (d) perceived financial barrier; (e) fear of a young infant’s fragility; (f) an elder’s contrary advice (“Did we grow up getting treatment? Why bring infants to the health facility?”); (g) distance; (h) husband’s refusal; (i) fear of injection that could be fatal in the presence of fancho ; and (j) belief that the child would recover without medicine (“The disease itself is the child”).” |
| Tegegne | Health care seeking behavior of parents with acute flaccid paralysis child | 2017 | World Health Organization | Journal article | Pan African Medical Journal | Cross-sectional survey | “Of 1299 families analyzed, 907(69.3%) of families with AFP child first went to health institutions to seek medical care, while. 398 (30.7%) of parents took their child first to other traditional sites, including holy water sites (11.8%), traditional healers (9.1%) and prayer places (5.4%). Over half of the parents with AFP child reported practicing home measures before first seeking health service from modern health institutions. Home measures (OR, 0.1202, 95% CI 0.0804-0.1797), decision by relatives (OR, 0.5595, 95% CI 0.3665-0.8540) and More than 10km distance from health facility (OR, 0.5962, 95% CI, 0.4117-0.8634) were significantly associated to first seeking health service from health institutions (p<0.05).” |
| World Health Organization/UNICEF | Behavioural, Socio-economic and Health Services Determinants of Immunization Service Utilization: A Community and Facility-Based Study in Ethiopia | Not specified | World Health Organization/United Nations Children’s Fund | Report | N/A | Cross-sectional surveys and qualitative interviews and focus group discussions | “The finding of this study identified that most of the barriers that stand against immunization practice were related to communication. This calls for improving the capacity, skills and knowledge of different actors including the community based health workers and volunteers. To this effect, a well designed training that addresses two major themes should be implemented. First, community based workers, community volunteers, and community based institutions need to be equipped with appropriate level and depth of information about immunization. Second, training should be given for health workers, especially for the facility and community based health workers and agents on interpersonal communication skills, counseling and problem solving skills to capacitate them so that they can deal with fear of side effects and misconceptions among immunization beneficiaries. “ |
